# The complete mitochondrial genome of a cryptic amphipod species from the *Gammarus fossarum* complex

**DOI:** 10.1080/23802359.2016.1275844

**Published:** 2017-01-11

**Authors:** Jan Niklas Macher, Florian Leese, Alexander Martin Weigand, Andrey Rozenberg

**Affiliations:** a Aquatic Ecosystem Research, Faculty of Biology, University of Duisburg-Essen, Universitätsstraße 5, Essen, Germany;; b Centre for Water and Environmental Research, University of Duisburg-Essen, Universitätsstraße 2, Essen, Germany;; c Beja's Lab, Faculty of Biology, Technion – Israel Institute of Technology, Haifa, Israel

**Keywords:** Mitogenome, amphipods, Gammaridae, cryptic species, Crustacea, biomonitoring

## Abstract

The freshwater amphipod *Gammarus fossarum* is widely distributed throughout Europe and an important species for stream biomonitoring. It is known to consist of several cryptic species. We here report the complete mitochondrial genome of *G. fossarum* clade 11/type B with a length of 15,989 bp, encoding for 13 protein-coding genes, 22 tRNA genes, and 2 rRNA genes. Protein-coding and ribosomal genes have a similar arrangement as in other gammarid amphipods. A phylogenetic analysis clarifies the placement of *G. fossarum* within the Gammaridae.

Amphipod crustaceans comprise a diverse taxon whose taxonomy is intensively studied (e.g. Myers & Lowry 2003; Hou & Sket 2016). The genus *Gammarus* is especially speciose and species are essential components of freshwater ecosystems, often forming important trophic links (MacNeil et al. 1997). Molecular studies revealed the common European *Gammarus fossarum* to be a species complex (Weiss et al. 2014), a widespread phenomenon in *Gammarus* (e.g. Mamos et al. 2016; Katouzian et al. 2016). Mitogenomes can help resolving amphipod phylogeny and diversity. Furthermore, mitogenomes are essential for future biomonitoring programmes using next-generation molecular tools (Crampton-Platt et al. 2016).

The sequenced specimen was collected in the Grundbächle (47.83N/7.93E) and belongs to *G. fossarum* clade 11/type B (Weiss & Leese 2016). It is stored in the UDE collection (accession number Gf_BF_S2). DNA was extracted as in Weiss and Leese (2016). A standard shotgun genomic library was sequenced on HiSeq 2000 (Otogenetics, USA), with 84,504,354 pairs of 101-nt reads obtained. Raw reads were trimmed with trimmomatic (v.0.22, Bolger et al. 2014) and mitochondrial reads were sampled with a blastn search against amphipod mitogenomes. The resulting data were assembled using MIRA (v.4.0, Chevreux 2005) and SOAPdenovo (v.2.04, Luo et al. 2012). Read re-mapping with bowtie2 (v.2.2.8, Langmead & Salzberg 2012) yielded 9095 read hits with a per-base coverage of 56.3. All three methods produced the same consensus sequence. Genes were annotated with MITOS WebServer (Bernt et al. 2013), and gene boundaries were refined manually. The complete circular mitogenome was 15,989 bp long (33.2% A, 22.0% C, 12.9% G, and 32.0% T) encompassing 13 protein-coding genes, 22 tRNA genes, 2 rRNA genes, and a ∼1500 bp AT-rich control region. The gene order appeared identical to that of *G. duebeni* (Krebes & Bastrop 2012).

For phylogenetic reconstruction, in addition to *G. fossarum* nine closely related species and two outgroups were taken. The mitochondrial genes were obtained from annotated mitochondrial genomes (NCBI GenBank), unannotated genome or transcriptome assemblies (NCBI WGS and TSA) and from raw transcriptome data assembled anew (NCBI SRA) (Figure 1). The dataset included 13 protein-coding genes (full-length except two cases), for each of which translation alignments were obtained with MAFFT (v.7.017, Katoh & Standley 2013). After manual trimming and concatenation, the resulting DNA alignment encompassed 10,863 nucleotides corresponding to 3621 amino acids. Four phylogenetic trees were produced using RAxML (v.8.2.9, Stamatakis 2014) and MrBayes (v.3.2.6, Ronquist & Huelsenbeck 2003) based on DNA and amino acid alignments with substitution models and partition schemes chosen with PartitionFinder (v.2.0.0, Lanfear et al. 2016).

**Figure 1.**
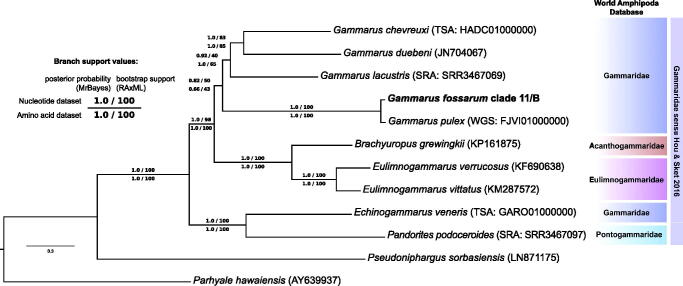
**The** Bayesian inference phylogenetic tree based on nucleotide alignment of 13 mitochondrial protein-coding genes for *Gammarus fossarum* (clade 11/type B) and 11 other amphipods. Both the current taxonomic status and the opinion of Hou and Sket (2016) are shown.

*Gammarus fossarum* clade 11/B forms a monophylum with other *Gammarus* species, rendering the genus monophyletic, albeit without a strong branch support (Figure 1). This stands in contrast to the findings by Hou et al. (2014). The Gammaridae as a whole are nevertheless paraphyletic in relation to the other related families, supporting the broader concept of the family proposed by Hou & Sket (2016).

The mitochondrial genome of *G. fossarum* clade 11/B was deposited in GenBank under the accession number KY197961. Extended methods description and sequence data are available at figshare (DOI:10.6084/m9.figshare.4487912).
